# *De novo* characterization of *Larix gmelinii* (Rupr.) Rupr. transcriptome and analysis of its gene expression induced by jasmonates

**DOI:** 10.1186/1471-2164-14-548

**Published:** 2013-08-13

**Authors:** Lina Men, Shanchun Yan, Guanjun Liu

**Affiliations:** 1 State Key Laboratory of Tree Genetics and Breeding, Northeast Forestry University, No. 26 Hexing Road, Harbin 150040, P. R. China

## Abstract

**Background:**

*Larix gmelinii* is a dominant tree species in China’s boreal forests and plays an important role in the coniferous ecosystem. It is also one of the most economically important tree species in the Chinese timber industry due to excellent water resistance and anti-corrosion of its wood products. Unfortunately, in Northeast China, *L. gmelinii* often suffers from serious attacks by diseases and insects. The application of exogenous volatile semiochemicals may induce and enhance its resistance against insect or disease attacks; however, little is known regarding the genes and molecular mechanisms related to induced resistance.

**Results:**

We performed *de novo* sequencing and assembly of the *L. gmelinii* transcriptome using a short read sequencing technology (Illumina). Chemical defenses of *L. gmelinii* seedlings were induced with jasmonic acid (JA) or methyl jasmonate (MeJA) for 6 hours. Transcriptomes were compared between seedlings induced by JA, MeJA and untreated controls using a tag-based digital gene expression profiling system. In a single run, 25,977,782 short reads were produced and 51,157 unigenes were obtained with a mean length of 517 nt. We sequenced 3 digital gene expression libraries and generated between 3.5 and 5.9 million raw tags, and obtained 52,040 reliable reference genes after removing redundancy. The expression of disease/insect-resistance genes (e.g., phenylalanine ammonialyase, coumarate 3-hydroxylase, lipoxygenase, allene oxide synthase and allene oxide cyclase) was up-regulated. The expression profiles of some abundant genes under different elicitor treatment were studied by using real-time qRT-PCR.

The results showed that the expression levels of disease/insect-resistance genes in the seedling samples induced by JA and MeJA were higher than those in the control group. The seedlings induced with MeJA elicited the strongest increases in disease/insect-resistance genes.

**Conclusions:**

Both JA and MeJA induced seedlings of *L. gmelinii* showed significantly increased expression of disease/insect-resistance genes. MeJA seemed to have a stronger induction effect than JA on expression of disease/insect-resistance related genes. This study provides sequence resources for *L. gmelinii* research and will help us to better understand the functions of disease/insect-resistance genes and the molecular mechanisms of secondary metabolisms in *L. gmelinii*.

## Background

Larches (*Larix* spp. Mill.) are major tree species of Northeast Asia boreal forests [1]. The role of *Larix* spp. in the boreal forest ecosystem is noteworthy because of its ability to grow on poor soils and on steep slopes prone to erosion and mass wasting, and its ability to withstand extremely cold winter temperatures while tolerating periodic summer-time forest fires common to the region [2,3]. *Larix gmelinii* (Rupr.) (Dahurian larch) populates large, climatically diverse areas, and is one of the most economically and ecologically important tree species in Northeast China, due to its excellent water resistance and anti-corrosion properties (acid and alkali resistance). For its long life span and extreme diversity of growth conditions, *L. gmelinii* is ravaged by a large number of herbivorous insects and pathogenic fungi, and a number of specialized insects are causing substantial losses to both natural and plantation forests, in particular during outbreak periods.

Genomic sequences available for *L. gmelinii* are scarce. Currently (Jul. 24th, 2013), there are 899 ESTs and 262 nucleotide sequences available on NCBI for *L. gmelinii*.

The transcriptome is a total set of transcripts, mRNA and non-coding RNA in a population of cells for all expressed genes. The transcriptome analysis lays the foundation for gene structure and function research. Next generation sequencing (NGS) technologies such as RNA-Seq using Illumina platform have applications in many research fields including re-sequencing, micro-RNA expression profiling, DNA methylation and have been utilized extensively for model [4-12] and non-model [13-19] organisms. To date, NGS has been used to sequence gymnosperm transcriptomes including, *Pinus taeda*, *Picea sitchensis* and *Taxus*[20-22]. Despite the obvious potential, researchers have not utilized NGS methods to study *L. gmelinii* disease/insect-resistance gene expression. We adopted the Solexa Illumina sequencers platform in sequencing the *L. gmelinii* to develop genomic resources for studies. Sequencing the transcriptome of *L. gmelinii* will provide a repository of genomic sequences for researchers studying *L. gmelinii* and improve our understanding of the functions/mechanisms of disease/insect-resistance genes and secondary metabolites in *L. gmelinii*.

Jasmonic acid (JA) and its volatile derivative methyl jasmonate (MeJA), collectively called jasmonates, are plant stress hormones that have regulatory functions as signalling molecules in higher plant development and adaptation to environmental stress [23-30]. Jasmonates activate host defense responses against a broad spectrum of herbivores. Although it is well established that JA controls the expression of a large set of target genes in response to tissue damage, very few gene products have been shown to play a direct role in reducing herbivore performance [31,32]. Exogenous application of MeJA results in major reprogramming of defensive gene expression in plants, inducing induction of chemical defenses, and effects similar to the ones induced by mechanical or herbivore damaged plants [27,28,32]. A number of genes that are known to be involved in plant stress responses are induced by JA treatment. JA induces the expression of genes encoding proteinase inhibitors (PIN), which are involved in the protection of plants from insect damage [33]. cDNA macro-array analysis revealed that MeJA treatment induced expression of several genes involved in JA biosynthesis, oxidative burst, stress-related and programmed cell death [34-40]. While defensive-associated signal has been extensively studied in angiosperms [41-43], it is a little-known in conifers. In conifers, biochemical changes induced by application of MeJA are similar to those induced by wounding, insect herbivore feeding, and pathogen invasion [28,44-47]. In our research presented here we studied expression of the genes related to induced resistance from *L. gmelinii* treated with JA and MeJA. Prior to this report, changes in protective enzymes, secondary metabolites and volatile compounds in *L. gmelinii* needles induced by JA or MeJA treatment were studied by our team [48,49]. In order to understand the impact of JA and MeJA on *L. gmelinii* at the transcriptional-level, the analysis of differentially expressed genes (DEGs) using digital gene expression was conducted. The differential gene expression profiles might provide an invaluable resource for the investigation of molecular mechanisms in *L. gmelinii* disease/insect-resistance and their potential defensive signals.

## Results and discussion

### High-throughput transcriptome sequencing and reads assembly

*L. gmelinii* gene expression profiles were constructed from cDNA synthesized from plants treated with JA and MeJA, and then sequenced with the Illumina sequencing platform. We obtained 25,977,782 short reads by sequencing. Q20 percentage (sequencing error rate <1%) and GC content were 94.97% and 46.28%, respectively. These reads were assembled with SOAPdenovo [9]. Our results revealed 545,211 contigs, the longest assembled sequences containing no Ns. By mapping reads back to contigs and combining paired-end information, contigs were linked into scaffolds. 92,511 scaffolds were assembled. Unknown bases were filled in with Ns. After filling gaps in scaffolds by using paired-end reads, we obtained 51,157 unigenes (Additional file 1) with mean unigene size being 517 nucleotides (nt) (Table 1). Additional file 2 indicates that the number of sequences with matches in the non-redundant (Nr) NCBI nucleotide database is greater for the longer assembled sequences.

**Table 1:** **Summary for the *Larix gmelinii *transcriptome**

	
Total number of reads	25,977,782
Total base pairs (nt)	2,338,000,380
Average read length (nt)	90
Total number of contigs	545,211
Mean length of contigs (nt)	130
Total number of scaffolds	92,511
Mean length of scaffolds (nt)	348
Total number of unigenes	51,157
Mean length of unigenes (nt)	517
Sequences with E-value<10^-5^	32,445

### Functional annotation

#### ***Annotation of predicted proteins***

Protein functions can be predicted from annotation of the most similar proteins in Nr, Swiss-Prot, KEGG and COG databases. We matched unigene sequences against two protein databases, Nr and Swiss-Prot, and obtained 32,445 and 21,092 unigenes respectively (Table 2). Distinct gene sequences were first searched using BLASTx against the Nr database using a cut-off E-value of 1.0E-5. The number of identified genes (32,445) based on the above cut-off value is not large because of the relatively short length of distinct gene sequences (mean size of 348 bp) and lack of genomic information on *L. gmelinii*.

**Table 2:** All-in-one list of annotations

**Database**	**Number**	**% of Total unigenes**
Nr (E-value<10^-5^)	32,445	63.42%
Swiss-Prot	21,092	41.23%
COG	9,920	19.39%
GO	13,317	26.03%
KEGG	14,462	28.27%
Total unigenes	51,157	100%

The proportion of sequences with matches in the Nr database was greater among the longer assembled sequences than shorter sequences. Over 98% of sequences longer than 2,000 bp or between 1,000 to 2,000 bp, matched gene sequences in the Nr database. The matching efficiency of the sequences between 1,000 to 2,000 bp were 98.1%, and those longer than 2,000 bp were 99.2%. For sequences between 500 to 1,000 bp, the matching efficiency decreased to 84.3%. For those ranging from 200 to 500 bp matching efficiency decreased to 51.9% (Additional file 3).

The E-value distribution of the top hits in the Nr databases showed that 27% of the mapped sequences have a strong homology (smaller than 1.0E-50), whereas 73% of the homolog sequences ranged between 1.0E-5 to 1.0E-50. The similarity distribution had a comparable pattern with 10% of the sequences having a similarity higher than 80%, while 49% of the hits had a similarity ranging from 51% to 80%. For genus distribution, 27.49% of the distinct sequences had top matches (first hit) with sequences from *Arabidopsis*, followed by the *Oryza* (21.77%), *Picea* (11.56%), *Zea* (5.16%) and *Populus* (3.78%). We matched unigene sequences against the Nr database and 32.96% of these unigene sequences matched to model organisms (*Arabidopsis, Nicotiana* and *Populus*) (Additional file 4).

### Clusters of orthologous groups (COG) classification, Gene ontology (GO) and KEGG

Overall, 9,920 sequences out of 32,445 Nr hits, had a COG classification (as shown in Table 2). Among the 25 COG categories, 2,693 genes (27.15%) fell into the cluster for general function prediction only. 1,316 gnes (13.27%) fell into the COG transcription category. 1,305 genes (13.16%) were categorized as having a role in the posttranslational modification, protein turnover and chaperones COG group. 1,250 (12.60%) of genes fell into the replication, recombination and repair COG group. Cell motility, extracellular structures and nuclear structure COG groups contained the fewest genes (Additional file 5).

**Figure 1:**
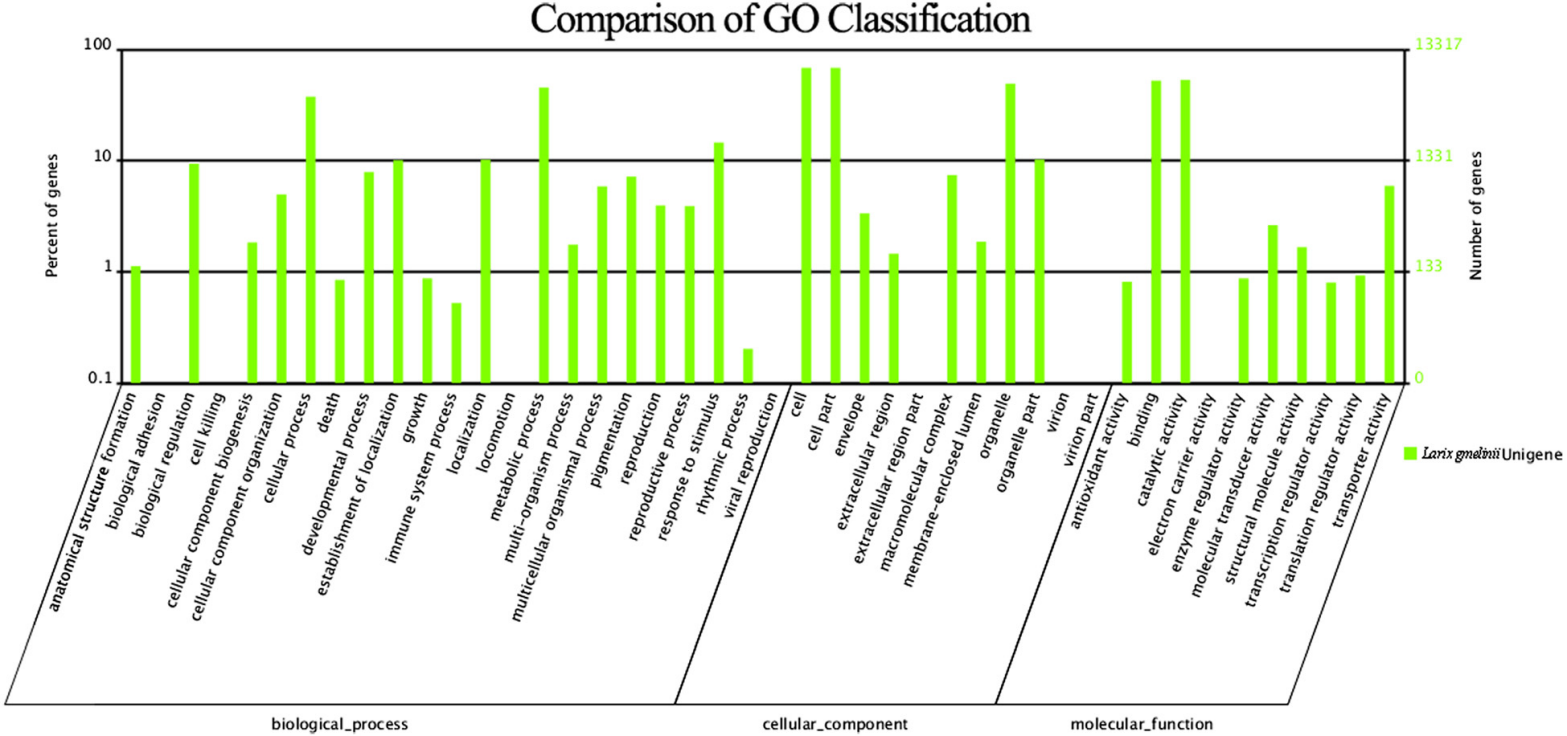
**Histogram presentation of gene ontology classification.** The results are summarized in three main categories: biological process, cellular component and molecular function. The left y-axis indicates the percentage of genes for each function belonging to a main category of genes in that main category; the right y-axis indicates the correspondent number of genes.

We obtained Gene Ontology (GO) functional annotation according to the Nr annotation. Based on sequence homology, 13,317 sequences were categorized into 44 functional groups (Figure 1). We found in each of the three main categories (biological process, cellular component and molecular function) of the GO classification that metabolic process, cell part & cell and catalytic activity are dominant functions, respectively; we also noticed a high-percentage of genes from the categories binding, organelle and cellular process, and only a few genes from the functions of locomotion, cell killing, virion and virion part (Figure 1).

In total, we assigned 14,462 sequences to 119 KEGG pathways as shown in Table 2. The pathways most represented by the unique sequences were metabolic pathways (3,381 members), biosynthesis of secondary metabolites (1,935 members), spliceosome (847 members), phenylpropanoid biosynthesis (513 members), starch and sucrose metabolism (423 members) and flavonoid biosynthesis (316 members). These annotations provide a valuable resource for investigating specific processes, functions and pathways in *L. gmelinii* genes.

We believe that genes in the KEGG categories metabolic pathways and starch and sucrose metabolism play a significant role in plant growth and development. Pathways such as flavonoid and phenylpropanoid biosynthesis are important in plant stress resistance. Phenylpropanoids are a group of secondary plant metabolites derived from phenylalanine and function as structural and signaling molecules. Phenylalanine is first converted to cinnamic acid by deamination, which is followed by hydroxylation and several methylation steps to generate coumaric acid and other acids with a phenylpropane (C6-C3) unit. Reduction of the CoA-activated carboxyl groups of these acids result in the synthesis of corresponding aldehydes and alcohols. The alcohols are called monolignols, and are starting components for the biosynthesis of lignin. These simple phenolic compounds are important in plant defense against fungi and herbivorous insects. As a result, phenylpropanoid metabolic pathways play an important role in plant growth, development and defense responses against pathogen and herbivore attacks [50].

### Protein Coding Region (CDS) Prediction

In total, 32,047 and 2,771 unigenes were predicted by BLASTx and ESTScan, respectively. The histogram as seen in Additional file 6 showed the length distribution of CDS predicted by BLAST and ESTScan. In general, as the sequence length increases, the number of CDS is gradually reduced. This is consistent with unigene assembly results [51].

### Digital gene expression (DGE) library sequencing

An immediate application of our transcriptome sequence data includes gene expression profiling from treatment with JA and MeJA. We used the DGE method which generates absolute rather than relative gene expression measurements and avoids many of the inherent limitations of microarray analysis. We sequenced three DGE libraries: Uninduced control (CK) vs. JA, CK vs. MeJA, and generated between 3.5 and 5.9 million raw tags for each of the three samples (Table 3). After removing the low quality reads, the total number of tags per library ranged from 3.3 to 5.6 million and the number of tag entities with unique nucleotide sequences ranged from 107,570 to 140,268 (Table 3). Heterogeneity and redundancy are two significant characteristics of mRNA expression. A small subset of mRNAs have very high abundance, while the majority of transcripts had a low level of expression. Therefore, the distribution of tag expression can be used to evaluate the normality of the DGE data. The distribution of total and distinct tags, different tag abundance categories showed similar patterns for all three DGE libraries (Additional file 7). Low-expression tags with copy numbers smaller than 10 occupied the majority of distinct tag distributions (Additional file 7). To evaluate the reproducibility of DGE library sequencing, we used Pearson correlation analysis for every two samples to indicate the reliability of our experimental results as well as operational stability (Additional file 8).

**Table 3:** Statistics of DGE sequencing

**Summary**		**CK**	**JA**	**MeJA**
Raw Data	Total	3508779	5959646	3508306
Raw Data	Distinct Tag	252431	405541	302418
Clean Tag	Total number	3366354	5682475	3308622
Clean Tag	Distinct Tag number	114240	140268	107570
All Tag Mapping to Gene	Total number	1215015	1915314	1179280
All Tag Mapping to Gene	Total % of clean tag	36.09%	33.71%	35.64%
All Tag Mapping to Gene	Distinct Tag number	47520	51376	38662
All Tag Mapping to Gene	Distinct Tag % of clean tag	41.60%	36.63%	35.94%
Unambiguous Tag Mapping to Gene	Total number	1202951	1900711	1166300
Unambiguous Tag Mapping to Gene	Total % of clean tag	35.73%	33.45%	35.25%
Unambiguous Tag Mapping to Gene	Distinct Tag number	47135	50986	38356
Unambiguous Tag Mapping to Gene	Distinct Tag % of clean tag	41.26%	36.35%	35.66%
All Tag-mapped Genes	number	19443	20411	17544
All Tag-mapped Genes	% of ref genes	37.36%	39.22%	33.71%
Unambiguous Tag-mapped Genes	number	19153	20114	17285
Unambiguous Tag-mapped Genes	% of ref genes	36.80%	38.65%	33.21%
Unknown Tag	Total number	2151339	3767161	2129342
Unknown Tag	Total % of clean tag	63.91%	66.29%	64.36%
Unknown Tag	Distinct Tag number	66720	88892	68908
Unknown Tag	Distinct Tag % of clean tag	58.40%	63.37%	64.06%

*CK* Control Check, *JA* jasmonic acid, *MeJA* methyl jasmonate.

### Mapping sequences to the reference transcriptome

To study the molecular events behind DGE profiles, we mapped tag sequences of the three DGE libraries to our reference database generated in the aforementioned Illumina sequencing, EST sequences and nucleotide sequences from NCBI. This reference database contains 51,157 unigene, 966 EST and 1,558 nucleotide sequences. After removing redundant genes, we obtained 52,040 reference genes including 40,948 genes with CATG sites and 123,601 reference tags. Between the 107,570 and 140,268 distinct tags generated from the Illumina sequencing of the three libraries, 38,662 to 51,376 distinct tags were mapped to a gene in the reference database (Table 3). Tags mapped to a unique sequence are the most critical subset of the DGE libraries as they can explicitly identify a transcript. Unique tags (Table 3) could unequivocally identify 39.22% (20,411) of the sequences in our transcriptome reference tag database. To confirm if the number of detected genes increases proportionally to the number of sequence reads (total tag number), a saturation analysis was performed. Additional file 9 shows a trend of saturation where the number of detected genes almost ceases to increase when the number of reads reaches 3 million. Next, the level of gene expression was determined by calculating the number of unambiguous tags for each gene and by normalizing this to the number of transcripts per million clean tags (TPM). As summarized in Additional file 10, results show that transcribed mRNA for the majority of genes was present in fewer than thirty copies and only a small proportion of genes were highly expressed.

### Distribution of DGE tags on genes

We found that approximately 80% of the tags mapped to a CTAG site (data not shown), this is probably due to the incomplete NlaIII digestion during library preparation and the usage of alternative polyadenylation and/or splicing sites [52]. Detection of multiple tags with high abundance for a predicted transcript indicates the reliability of the transcript sequence [53]. Furthermore, the information obtained from multiple tags per transcript is valuable for the verification of *ab initio* gene predictions.

### Changes in gene expression profile induced by different elicitor treatments

To identify a significant change in gene expression by different elicitor treatments, the differentially expressed tags between two treatment samples (JA and MeJA) were identified by an algorithm developed by Audic *et al*. [54]. 13,884 tags with significantly altered expression were detected between the CK and JA *L. gmelinii* libraries. Filtered with FDR≤0.001 and |log2Ratio|≥1, these tags were mapped to a total of 2,383 genes of which 600 were up-regulated and 1,783 were down-regulated (Additional files 11 and 12). In addition, a total of 13,623 tags with significantly altered expression were detected between CK and MeJA of *L. gmelinii* libraries, which mapped to 2,767 genes, 1,025 genes were up-regulated and another 1,742 were down regulated (Additional file 13). The total number of differentially expressed genes between CK and MeJA (2,767) is larger than that of CK and JA (2,383). Roughly the same number of down-regulated genes (1,783 and 1,742) were detected under the conditions produced by two elicitors. Furthermore, we analyzed the differentially expressed genes between every two sample DGEs (Additional file 14), and found that less than 40% of the differentially expressed genes are orphan sequences, with no homologues found in the NCBI database. Next, we analyzed the 20 most strongly expressed genes between the two treatments (CK vs. JA and CK vs. MeJA) and identified nine up-regulated genes and four down-regulated genes present in both treatments (Additional file 15). Therein some highly expressed genes are involved in many important biological pathways, for instance, the TPMs of Unigene16480 (Table 4) in JA and MeJA treated plants are 82.01 and 128.75, respectively, and are involved in six pathways, namely, “metabolic pathways”, “biosynthesis of secondary metabolites”, “flavonoid biosynthesis”, “phenylpropanoid biosynthesis”, “stilbenoid, diarylheptanoid and gingerol biosynthesis” and “phenylalanine metabolism”. These highly expressed genes probably have important biological functions and should be investigated in future studies (Table 4).

**Table 4:** Top 20 highly expressed genes related to pathways

**Unigene**	**Pathways**	
	**CK vs. JA**	**CK vs. MeJA**
Unigene16480	Metabolic pathways	Metabolic pathways
	Biosynthesis of secondary metabolites	Biosynthesis of secondary metabolites
	Flavonoid biosynthesis	Flavonoid biosynthesis
	Phenylpropanoid biosynthesis	Phenylpropanoid biosynthesis
	Stilbenoid, diarylheptanoid and gingerol biosynthesis	Stilbenoid, diarylheptanoid and gingerol biosynthesis
	Phenylalanine metabolism	Phenylalanine metabolism
Unigene41734	Metabolic pathways	Metabolic pathways
	Oxidative phosphorylation	Oxidative phosphorylation
	Phagosome	Phagosome
Unigene16812	Metabolic pathways	Metabolic pathways
	Carbon fixation in photosynthetic organisms	Carbon fixation in photosynthetic organisms
	Glyoxylate and dicarboxylate metabolism	Glyoxylate and dicarboxylate metabolism
Unigene43815	Metabolic pathways	
	Linoleic acid metabolism	
	alpha-Linolenic acid metabolism	
Unigene46798*		Pyruvate metabolism
Unigene13715*		Ribosome

* Down-regulated highly express genes.

### Functional annotation of differentially expressed genes

To understand the functions of differentially expressed genes, functional GO enrichment analysis was executed in order to determine major biological functions of DEGs between the two treatment samples (JA and MeJA) (Additional file 16). We found that oxidoreductase activity was the most significantly enriched GO-term of molecular functions in DEGs between CK vs. JA and CK vs. MeJA. “Cytoplasmic part” was the most significantly enriched GO-term of “cellular component” and lack of the GO-term of “biological process” in DEGs of the sample CK vs. JA. By using functional GO enrichment analysis, the result showed that “plastid part” was the most significantly enriched GO-term of “cellular component”, and “cellular cell wall organization” of “biological process” was the same in DEGs for CK vs. MeJA sample. Different genes usually cooperate with each other to exercise their biological functions. Pathway-based analysis helps to understand a gene’s biological functions.

KEGG is the major public pathway-related database. We mapped all of the genes to terms in KEGG database, and compared this with the whole transcriptome to search for enriched genes involved in metabolic or signal transduction pathways. Among the genes with KEGG pathway annotations, 835 differentially expressed genes were identified in CK and JA libraries, and 932 in CK and MeJA libraries. Pathway enrichment analysis identifies significantly enriched metabolic or signal transduction pathways in DEGs by comparison with the transcriptome background. Pathways with Qvalue≤0.05 are significantly enriched in DEGs. These results showed that 11 pathways were significantly enriched in DEGs of CK and JA, including “metabolic pathways”, “protein processing in endoplasmic reticulum”, and so on (Additional file 17). Notably, specific enrichment of genes was observed for pathways involved in energy and organic compound biosynthesis or metabolism, such as the oxidative phosphorylation, photosynthesis, biosynthesis of secondary metabolites and pyruvate metabolism (Additional file 17).

**Figure 2:**
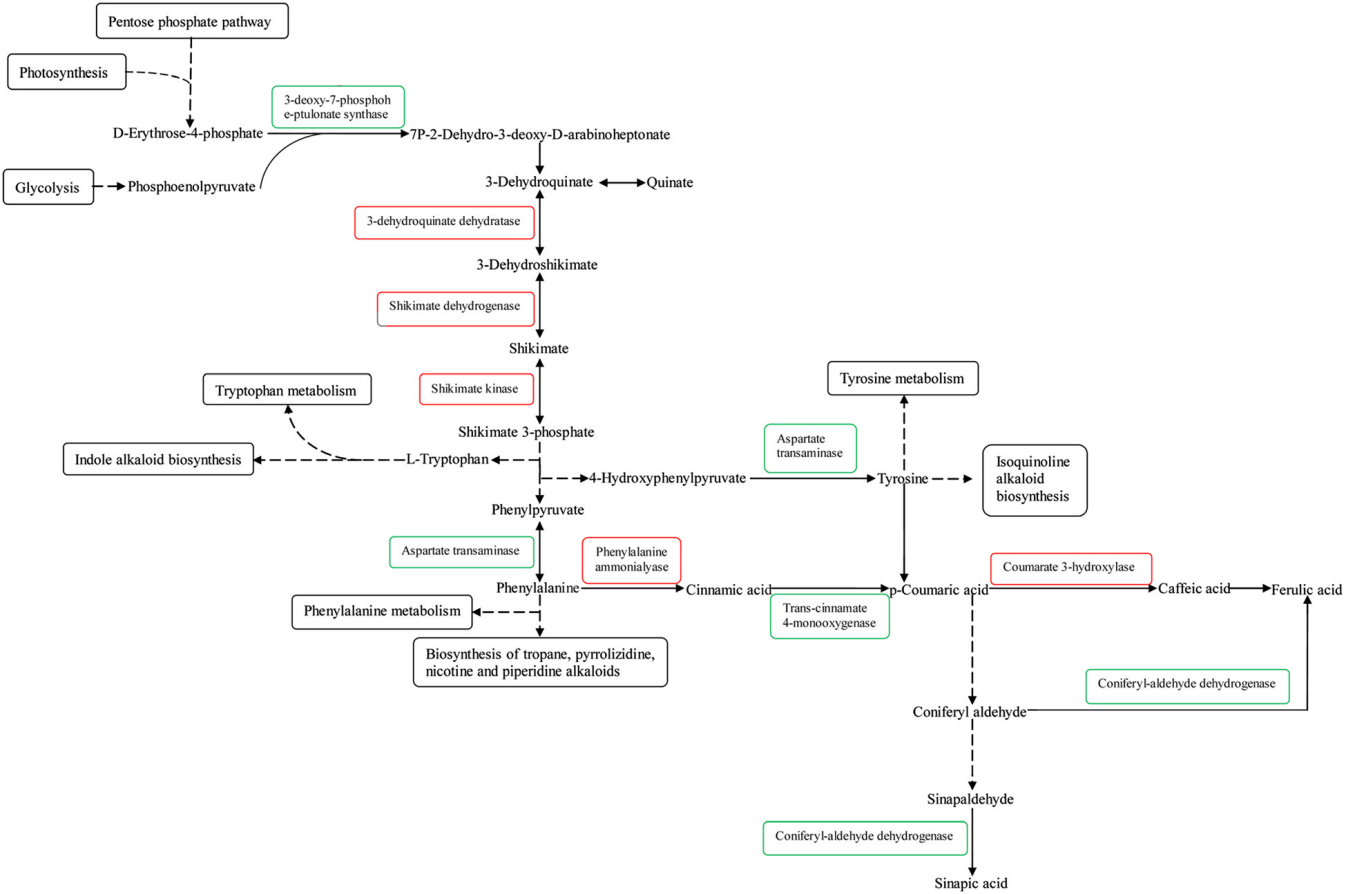
**Phenylalanine, tyrosine and tryptophan biosynthesis pathway induced by MeJA treatment.** Black borders: Pathway; up-regulated genes are marked with red; down-regulated genes are marked with green.

Upon wounding, fungal infection or insect attack, conifer trees respond with lesion formation, cell death and the accumulation of constitutive and induced phenolics and terpenoids in the affected areas [55]. This is hypersensitive response and the release of toxic chemicals may restrict and possibly kill invading insects and fungal pathogens. The induced activation of polyphenolic parenchyma and traumatic resin ducts, which are formed in response to the attacks, further enhance conifer defense capacity against the current threat and additional attacks [56]. Several genes involved in phenylpropanoid pathway produce lignin, flavonoids and other phenylpropanoid phytoalexins [57,58], these genes in the phenylpropanoid pathway of conifers and their involvement in defense mechanisms have also been characterized [59-64]. Many phenolic compounds play an important function/role in plant defense against attacks by fungi and herbivorous insects and are synthesized through pathways, most notably, the shikimic acid pathway, is the most pivotal pathway. Phenylalanine, tyrosine and tryptophan are also synthesized through the shikimic acid pathway. Phenolic compounds are produced from these amino acids via a series of hydroxylation, methylation and dehydration reactions through the phenylpropanoid synthesis pathway. The phenylpropanoid pathway also plays a role is synthesis of defensive compounds [65]. Phenolic secondary metabolites have been proposed to play a role in conifer defence against pathogens and potentially insects as well [56,66,67]. We noticed that in the phenylalanine biosynthesis pathway, the gene expression of 3-dehydroquinate dehydratase and shikimate dehydrogenase was up-regulated by MeJA treatment and was down-regulated by JA treatment. The gene expression of aspartate transaminase was down-regulated simultaneously by JA and MeJA treatment. The effect of JA was stronger than MeJA (Figure 2). There were 14 genes expressed in the phenylpropanoid biosynthetic pathway, which were found by sequencing the *L. gmelinii* transcriptome from needles induced by JA or MeJA. The 14 genes were phenylalanine ammonia-lyase (PAL), cinnamate-4-hydroxylase (C4H), *p*-coumarate-3-hydroxylase (C3H), ferulate-5-hydroxylase (F5H), 4-coumarate CoA ligase (4CL), caffeoyl-CoA O-methyltransferase (CCoAOMT), cinnamoyl-CoA reductase (CCR), cinnamyl alcohol dehydrogenase (CAD), beta-glucosidase, peroxidase, shikimate O-hydroxycinnamoyltransferase, coniferyl-alcohol glucosyltransferase, coniferyl-aldehyde dehydrogenase and sinapate 1-glucosyltransferase. This result is consistent with the involvement of this pathway in *Pinus sylvestris* defense against *Heterobasidium annosum* and in *Picea sitchensis* (sitka spruce), against mechanical wounding or feeding by *Choristoneura occidentalis* or *Pissodes strobi*[68,69]. Most of the annotated transcripts likely have a different function, among which the genes were identified only in angiosperms to be involved in the biosynthetic pathway [70]. Among the 14 genes, 8 can be found in the phenylpropanoid biosynthetic pathway of *P.sitchensis* phloem induced by fungus or insect feeding or mechanical damage [69]. The 2 genes of hydroxycinnamoyl-CoA shikimate/quinate hydroxycinnamoyltransferase (HCT) and caffeic acid O-methyltransferase (COMT) were not expressed in induced *L. gmelinii*, but were expressed in induced *P. sitchensis*. The 6 genes of beta-glucosidase, peroxidase, shikimate O-hydroxycinnamoyltransferase, coniferyl-alcohol glucosyltransferase, coniferyl-aldehyde dehydrogenase and sinapate 1-glucosyltransferase were expressed in induced *L. gmelinii*, but were not detected in the induced *P. sitchensis*[69]. In gymnosperms from different genera, the enzymes involved in the phenylpropanoid pathway may induced by a stimulus. Different stimuli may cause expression of different enzymes.

The genes of PAL and enzymes which catalyze formation of coumaric acid, caffeic acid, ferulic acid and sinapic acid, such as trans-cinnamate 4-monooxygenase, coumarate 3-hydroxylase and coniferyl-aldehyde dehydrogenase, were expressed in both induced *L. gmelinii* and *P. sitchensis*[69]. It is similar that the induction trend of the phenylpropanoid pathway by JA or MeJA treatment and in spruce exposed to herbivory, and highlights common response of the phenylpropanoid pathway to stresses in conifer. PAL catalyzes the first reaction of the phenylpropanoid pathway and has been shown to increase in response to stress from wounding or fungal elicitors in pine cell cultures and trees [50,60,71]. PAL is also associated with the initiation of phenolic metabolism including biosynthesis of lignans and lignins [72]. We also found that the expression of PAL gene was significantly higher in *L. gmelinii* treated with JA or MeJA than the control, and the expression was greater when treated with MeJA than JA. This result was consistent with our previous results [48,49] and similar to the results of MeJA-treated *Arabidopsis*[40], and treated hairy roots of *Daucus carota*[73]. PAL is highly expressed in poplar leaves and expression increases after insect feeding [74]. The invasion of the fungus *Ceratocystis polonica* causes induced activation with enrichment of PAL at the plasma membrane of phenolic PP cells in *Picae abies*[75]. These previous studies are consistent with our results. JA, MeJA and fungal infection are able to increase expression of PAL.

The gene expression of trans-cinnamate 4-monooxygenase and coniferyl-aldehyde dehydrogenase which catalyze the synthesis of coumaric acid and ferulic acid in *L. gmelinii* was down-regulated simultaneously after being induced by JA and MeJA*.* Expression of coumarate 3-hydroxylase, which is required for synthesis of caffeic acid, was up-regulated in MeJA treatment samples. In JA treated samples coumarate 3-hydroxylase is down-regualted, which contrasts with previous results from our team that the caffeic acid, ferulic acid and coumaric acid content increases in induced *L. gmelinii* needles [48,49]. Future studies are required to determine if post-transcriptional level is involved (Additional file 18).

Several studies indicate that glucosinolate and its degradation products play an important role in plant resistance against herbivores, insects and pathogens [76-78]. In the glucosinolate biosynthesis pathway, up-regulated genes responsible for transferase activity and transferring glycosyl groups or glucosyl transferase were induced by JA and MeJA treatment. Stronger induction occurred in plants treated with JA.

**Figure 3:**
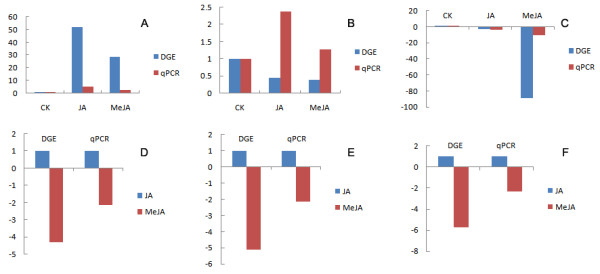
**qRT-PCR validation of DGE tag data.** The x-axis indicates treatment method. The y-axis indicates relative expression level. The following selection of genes were tested with their description. **(A)** phenylalanine ammonialyase (Unigene 46956), **(B)** cinnamate 4-hydroxylase (Unigene 47342). **(C)** 4-coumarate-CoA ligase (Unigene 11155), **(D)**  trans-cinnamate 4-monooxygenase (Unigene 20026), **(E)** caffeic acid 3-O-methyltransferase (Unigene 15709), **(F)** caffeoyl-CoA O-methyltransferase (Unigene 16480).

It is well known that the alpha-linolenic acid metabolic pathway is involved in JA synthesis and is also a signaling pathway that induces the expression of protease inhibitors [79] and other plant defense genes [80-82]. Alpha-linolenic acid metabolic pathway is octadecanoid pathway of plant in response to those induced by wounding, insect herbivore attack and pathogen invasion feeding revealed of putatively involved. Gene expression analysis of *P. sitchensis* in response to *P. strobi* feeding revealed an increased expression of genes putatively involved in the octadecanoid pathway [44,69]. The ability of MeJA to induce defense responses, similar to those elicited by wounding, insect feeding, and fungal inoculations, is consistent with the role for octadecanoid signaling in induced conifer chemical defense. Lipoxygenase (LOX) is a key enzyme in this pathway [83,84], allene oxide synthase (AOS) is the first enzyme of the LOX synthesis pathway, whereas allene oxide cyclase (AOC) is the key enzyme of JA biosynthesis. We found that even though the gene expression of LOX, AOS and AOC in *L. gmelinii* is up-regulated by both two elicitor treatments, again, the quantity of expression in JA treated plants is greater than that in MeJA. LOX is highly expressed after insects feed on *Nicotiana tabacum*, and LOX activity in *Populus simonii* × *P. pyramidalis* ‘Opera 8277’ leaves increased after *Clostera anachoreta* feeding [74]. The relative expression of LOX was significantly higher in *Arabidopsis* and *Solanum lycopersicum* treated with MeJA than the control [36,38-40]. Similarly, these gene expressive levels were increased after MeJA treatment in *P. sitchensis*[44]. These findings in angiosperm and *P. sitchensis* are consistent with our results. In order to verify a subset of the DGE tag data by a third independent technology, quantitative reverse transcriptase PCR (qRT-PCR) analysis were conducted. The 6 genes expressed in phenylpropanoid pathways under different elicitors treatment were studied by using real-time qRT-PCR. The results showed a similar direction between DGE and qRT-PCR in induced by JA and MeJA (Figure 3). qRT-PCR analysis confirmed the direction of changes detected by DGE analysis.

## Conclusions

In this study, we sequenced the *L. gmelinii* transcriptome and used it to study gene expression of disease/insect-resistance genes in trees induced with JA or MeJA. Several disease/insect-resistance genes were identified. Part of these defense genes were also found in *P. sitchensis* induced by biotic and abiotic stress [44,69]. A few genes associated with induced chemical defense against insects were recorded in GenBank of spruce, larch and other gymnosperms (e.g. *Pinus*, *Pseudotsuga*, *Selaginella*, *Ginkgo*, *Taxus* and *Abies*). Our transcriptome and digital gene expression emphasized the potential importance of JA and MeJA on *L. gmelinii* induced defense, as well as the role of phenolic secondary metabolite pathways, specifically the phenylpropanoid biosynthesis pathway.

A single run produced more than 51,157 unigene sequences, from which 32,445 sequences had a BLAST result based on E-value less than 1.0E-5. These findings provide a substantial contribution to the gene sequence resources for *L. gmelinii* gene research and will likely accelerate research of disease/insect-resistance related genes on *L. gmelinii*. The present digital gene expression library will guide in the selection of genes for their further functional characterization.

## Methods

### Plant materials and growth conditions

Our sample seedlings were provided by Forestry Administration of Jiagedaqi in Daxing’anling, Heilongjiang Province, P. R. China.

Three year old *L. gmelinii* seedlings (around 70 cm high) were planted in plastic pots (22 cm diameter, 30 cm height), and grown in soil, under a photoperiod of 12-h-light/12-h-dark, at 27 ± 1°C and 70 ± 10% relative humidity in a greenhouse [3,85].

### Plant treatments and RNA isolation

In July 2010, 15 *L. gmelinii* seedlings of similar condition and size were selected, and randomly divided into three groups. Each group contained 5 seedlings. Two months after the *L. gmelinii* needles sprouted, seedlings were treated with either jasmonic acid (JA), methyl jasmonate (MeJA), or aqueous acetone solution (control) by spraying the following aqueous acetone solutions: 0.1 mM JA in 0.1% acetone-distilled water solution; 0.1 mM MeJA in 0.1% acetone-distilled water solution and 0.1% acetone-distilled water solution [32,79,86]. Each seedling was sprayed with 20 ml of the treatment or control solution using a handheld sprayer [85]. *L. gmelinii* needles from the upper part of the seedling (circa 30 cm from the top) were sampled for total RNA isolation 6 h after being sprayed. Total RNA isolation was performed following the protocol outlined by Jaakola *et al*. [87].

### RNA library preparation for transcriptome analysis

RNA integrity was confirmed using the 2100 Bioanalyzer (Agilent Technologies) with a minimum integrated RNA value of 8.0. The samples for transcriptome analysis were prepared using Illumina's kit according to manufacturer recommendations. Beads with Oligo (dT) were used to isolate the poly (A) mRNA tails from total RNA (a mixture of RNA from CK, JA and MeJA at equal ratio). Following purification, fragmentation buffer was added for converting mRNA to short fragments. These short fragments were used as templates from which random hexamer-primers were used to synthesize first-strand cDNA. The second-strand cDNA was synthesized using buffer, dNTPs, RNaseH and DNA polymerase I. Short fragments were purified with the QiaQuick PCR extraction kit and resolved with EB buffer for end reparation and poly (A) addition. After that, the short fragments were connected with sequencing adapters and, after agrose gel electrophoresis, the suitable fragments were selected for the PCR amplification as templates. Finally, the library was sequenced using Illumina HiSeq™ 2000.

### Analysis of Illumina sequencing results

Transcriptome *de novo* assembly was carried out with the short reads assembling program-SOAPdenovo [9]. SOAPdenovo combines reads with a certain length of overlap to form longer fragments without N, which are called contigs. Then the reads are mapped back to contigs. With paired-end reads it is able to detect contigs from the same transcript as well as the distances between these contigs. Next, SOAPdenovo connects the contigs using N to represent unknown sequences between each two contigs and then creates scaffolds. The scaffold length was estimated according to average segment length of each pair of reads. Paired-end reads are used again for gap filling of scaffolds to produce sequences with the fewest Ns and cannot be extended on either end. These sequences are called unigenes. When multiple samples from the same species are sequenced, unigenes from each sample's assembly can be processed with sequence clustering software to reduce redundancy. In the final step, BLASTx alignment (E-value<0.00001) between unigenes and protein databases like Nr, Swiss-Prot, KEGG and COG was performed. The best alignment results were used to determine the sequence direction of unigenes. If results from databases conflicted with one another, a priority order of Nr, Swiss-Prot, KEGG and COG was used when deciding sequence direction of unigenes. When a unigene would not align to any database, ESTScan [88] was used to predict coding regions and determine sequence direction. For unigenes with sequence directions, we provided their sequences from 5' end to 3' end; for those without any direction we provided their sequences from assembly software. The transcriptome datasets are available at the NCBI BioProject with the accession number PRJNA171213.

### Digital gene expression library preparation and sequencing

Reagents and supplies were provided by the Illumina Gene Expression Sample Prep Kit and Illumina Sequencing Chip (flowcell). Instruments used included the Illumina Cluster Station and Illumina HiSeq™ 2000 System. Tag library preparation for the three *L. gmelinii* treatment samples (CK, JA and MeJA) were performed in parallel using the Illumina gene expression sample preparation kit. For each treatment, 6 μg of total RNA was adsorbed with Oligo (dT) magnetic beads to purify mRNA. Oligo (dT) was used as primer to synthesize the first and second-strand cDNA. The bead-bound cDNA was subsequently digested with restriction enzyme NlaIII, which recognizes and cuts at CATG sites. After digestion, the 5’ cDNA ends were washed away while the 3’ cDNA fragments remained bound to Oligo (dT) beads. The Illumina adaptor 1 was ligated to the sticky 5' ends of the digested bead-bound cDNA fragments. The junction of the Illumina adaptor 1 and the CATG site is the recognition site for endonuclease MmeI, which cuts at 17 bp downstream of the CATG site and produces tags with adaptor 1. After removing 3' fragments with magnetic bead precipitation, Illumina adaptor 2 was ligated to the 3' ends of tags. After 15 cycles of linear PCR amplification, 95 bp fragments were purified by 6% TBE PAGE Gel electrophoresis. After denaturation, the single-chain molecules were fixed to the Illumina Sequencing Chip (flowcell). Each molecule grows into a single-molecule cluster sequencing template through *in situ* amplification. Four types of fluorescently labeled nucleotides were added and sequenced by a sequencing by synthesis (SBS) method. Each tunnel will generate millions of raw reads with sequencing length of 35 bp.

### Analysis and mapping of DGE tags

Raw image data from sequencing was transformed by base calling into sequence data. Raw sequences have 3' adaptor fragments as well as a few low-quality sequences (Tags with unknown sequences 'N') and several impurities. Raw sequences are transformed into clean tags after data-processing and acquiring a virtual library containing all the possible CATG+17 base sequences of the reference gene sequences. All clean tags were mapped to the reference sequences and only 1 bp mismatch was allowed. Clean tags mapped to reference sequences from multiple genes were filtered. The remaining clean tags were designated as unambiguous clean tags. The number of unambiguous clean tags for each gene was calculated and then normalized to TPM (number of transcripts per million clean tags) [89,90]. Referring to "The significance of digital gene expression profiles" [54], we have used a rigorous algorithm to identify differentially expressed genes between two samples. P-value corresponds to the differential gene expression test. FDR (False Discovery Rate) is a method to determine the threshold P-value in multiple tests and analysis through manipulating the FDR value. Assuming that we have chosen R differentially expressed genes in which S genes actually have differential expression and the other V genes were false positives, the error ratio "Q=V/R" must stay below a cut-off (e.g. 1%) defined by the FDR [91]. We used "FDR≤0.001 and the absolute value of log2Ratio≥1" as the threshold to judge the significance of differential gene expression. More stringent criteria with smaller FDR and larger fold-change values can be used to identify DEGs. In gene expression profiling analysis, GO enrichment analysis of functional significance applies a hypergeometric test to map all differentially expressed genes to terms in the GO database and looks for significantly enriched GO terms in DEGs compared to the transcriptome background. Pathway enrichment analysis identifies significantly enriched metabolic pathways or signal transduction pathways in DEGs compared against the whole genome background.

### Real-time quantitative RT-PCR for the key genes in phenylalanine biosynthesis pathway

To investigate the expression of key genes in the phenylalanine biosynthesis pathway, the same RNA from each samples which was used in DGEs was analyzed with qPCR. The total RNA from each sample was reverse-transcribed using oligo-deoxythymidine as a primer in 10 μl. The cDNAs were diluted to 100 μl and used as a PCR template. qPCR was conducted using an MyiQ2 real-time PCR machine (Bio-Rad, Hercules, CA). Actin (GenBank number: AB602839.1) was used as an internal control. Among the 14 differentially expressed genes in the phenylalanine biosynthesis pathway, we choose 6, with contigs longer than 300 bp, which were suitable for designing the primers for qPCR (primers used are shown in Additional file 19). The reaction mixture (20 μl) contained 10 μl of SYBR Green Real-time PCR Master Mix (Toyobo), 0.5 μM of forward and reverse primers, and cDNA template (equivalent to 70 ng of total RNA). The amplification was executed with the following parameters: 94°C for 30 s; 45 cycles at 94°C for 5 s, 60°C for 15 s, 72°C for 10 s; and 80°C for 10 s for plate reading. A melting curve was generated for each sample to assess the purity of the amplified products. 5 biological replicates were performed with needles collected from each elicitor treatment, 3 technical replicates were performed on each needle. The expression levels were calculated from the threshold cycle according to the delta–delta CT method.

## Competing interests

The authors declare that they have no competing interests.

## Authors’ contributions

LNM designed and performed experiments, analyzed and interpreted the sequence data and drafted the manuscript. SCY and GJL conceived the study, and participated in the design and coordination and helped to draft the manuscript. All authors read and approved the final manuscript.

## Supplementary Material

Additional file 1 **Top BLAST hits from NCBI Nr and Swiss-Prot database.** BLAST results against the distinct sequences with a cut-off E-value above 10–5 are shown. Click here for file

Additional file 2 **Length distribution of *Larix gmelinii *Unigenes.** Histogram presentation of sequence-length distribution for significant matches that were found. The x-axis indicates sequence sizes from 200 nt to >3000 nt. The y-axis indicates the number of uingenes for every given size. The results of sequence-length matches (with a cut-off E-value of 1.0E-5) in the NCBI Nr databases are greater among the longer assembled sequences. Click here for file

Additional file 3 **Effect of query sequence length on the percentage of sequences for which significant matches were found.** The proportion of sequences with matches (with a cut-off E-value of 1.0E-5) in NCBI non-redundant NCBI nucleotide database is greater among the longer assembled sequences. Click here for file

Additional file 4 **Characteristics of homology search of Illumina sequences against the Nr databases.** (A) E-value distribution of BLAST hits for each unique sequence with a cut-off E-value of 1.0E-5. (B) Similarity distribution of the top BLAST hits for each sequence. (C) Species distribution is shown as a percentage of the total homologous sequences with an E-value of at least 1.0E-5 (we used the first hit of each sequence for analysis). (D) Model organisms distribution is shown as a percentage. Click here for file

Additional file 5 **Histogram presentation of clusters of orthologous groups (COG) classification.** These 9,920 sequences have a COG classification among the 25 categories. Click here for file

Additional file 6 **Length distribution of *Larix gmelinii *unigene for CDS predicted via BLAST and ESTScan.** (A) Length distribution of *Larix gmelinii* Unigene. BLAST. cds. fa; (B) Length distribution of *Larix gmelinii* Unigene. ESTScan. cds. fa. Click here for file

Additional file 7 **Distribution of total clean tags (A-C) and distinct clean tags (D-F) over different tag abundance categories.** Numbers in the square brackets indicate the range of copy numbers for a specific category of tags. For example, [2,5] means all the tags in this category have 2 to 5 copies. Numbers in the parentheses show the total tag copy number for all tag types in that category. Click here for file

Additional file 8 **Correlation analysis of DGE libraries.** The correlation between CK vs. JA and CK vs. MeJA libraries are shown. Dots in the figures indicate individual tag entities. Pearson correlation coefficients are shown in the upper left corner of each plot. Click here for file

Additional file 9 **Relationship between the number of detected genes and sequencing amount (total tag number).** All figures show a trend of saturation. When the sequencing amount reaches 3 million, the number of detected genes almost ceases to increase. Click here for file

Additional file 10 **Level of gene expression for each gene.** Gene expression level was determined by calculating the number of unambiguous tags for each gene and then normalizing to TPM (transcript copies per million tags). (A) control solutions CK; (B) JA-treatment; (C) MeJA-treatment. Click here for file

Additional file 11 **Changes in gene expression profile induced by different elicitor treatments.** The number of up-regulated and down-regulated genes between CK and JA; CK and MeJA is summarized. Click here for file

Additional file 12 **Differentially expressed genes between CK vs. JA.** TPM: transcript copies per million tags. Raw intensity: the total number of tags sequenced for each gene. FDR: false discovery rate. We used FDR<0.001 and the absolute value of |log2Ratio|≥1 as the threshold to judge the significance of gene expression difference. In order to calculate the log2Ratio and FDR, we used TPM value of 0.001 instead of 0 for genes that do not express in one sample. Click here for file

Additional file 13 **Differentially expressed genes between CK vs. MeJA.** TPM: transcript copies per million tags. Raw intensity: the total number of tags sequenced for each gene. FDR: false discovery rate. We used FDR<0.001 and the absolute value of |log2Ratio|≥1 as the threshold to judge the significance of gene expression difference. In order to calculate the log2Ratio and FDR, we used TPM value of 0.001 instead of 0 for genes that do not express in one sample. Click here for file

Additional file 14 **Summary of the most abundant genes expressed in CK, JA and MeJA.** TPM: number of transcripts copies per million tags. Click here for file

Additional file 15 **The top 20 most up-regulated and down-regulated genes between samples.** TPM: transcript copies per million tags. This table does not include genes that only expressed in one sample. Click here for file

Additional file 16 **Significantly enriched GO terms in DEGs.** GO terms with corrected-pvalue≤0.05 are significantly enriched in DEGs. Click here for file

Additional file 17 **Pathway significantly enriched of DEGs (CK vs. JA and CK vs. MeJA).** Pathways with Qvalue≤0.05 are significantly enriched in DEGs. Click here for file

Additional file 18 **Differential gene expression of Phenylpropanoid biosynthesis.** + means up-regulated; - means down-regulated; log2Ratio: multiples of differentially expressed. Click here for file

Additional file 19 qPCR primer sequences. Click here for file
