# Supplementary figures and images for: De novo characterization of Larix gmelinii (Rupr.) Rupr. transcriptome and analysis of its gene expression induced by jasmonates
Source: BMC Genomics. 2013 Aug 13;14:548. doi: 10.1186/1471-2164-14-548 (PMC3765852; doi:10.1186/1471-2164-14-548)

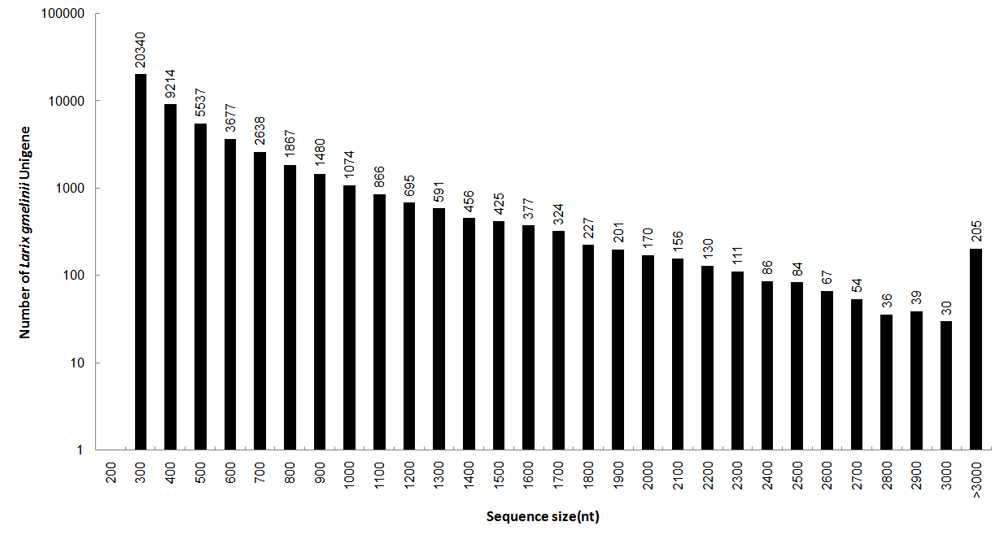

Supplement: Additional file 2 — Length distribution of Larix gmelinii Unigenes. Histogram presentation of sequence-length distribution for significant matches that were found. The x-axis indicates sequence sizes from 200 nt to >3000 nt. The y-axis indicates the number of uingenes for every given size. The results of sequence-length matches (with a cut-off E-value of 1.0E-5) in the NCBI Nr databases are greater among the longer assembled sequences. [file 1471-2164-14-548-S2.tiff]

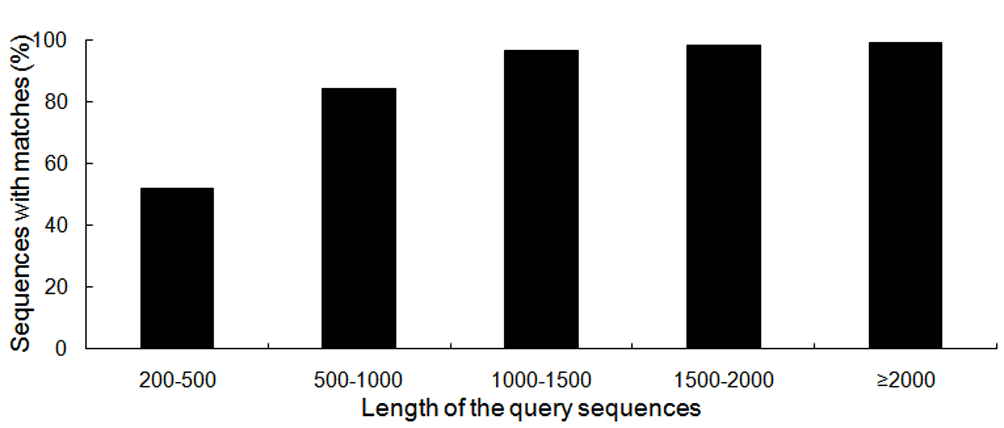

Supplement: Additional file 3 — Effect of query sequence length on the percentage of sequences for which significant matches were found. The proportion of sequences with matches (with a cut-off E-value of 1.0E-5) in NCBI non-redundant NCBI nucleotide database is greater among the longer assembled sequences. [file 1471-2164-14-548-S3.tiff]

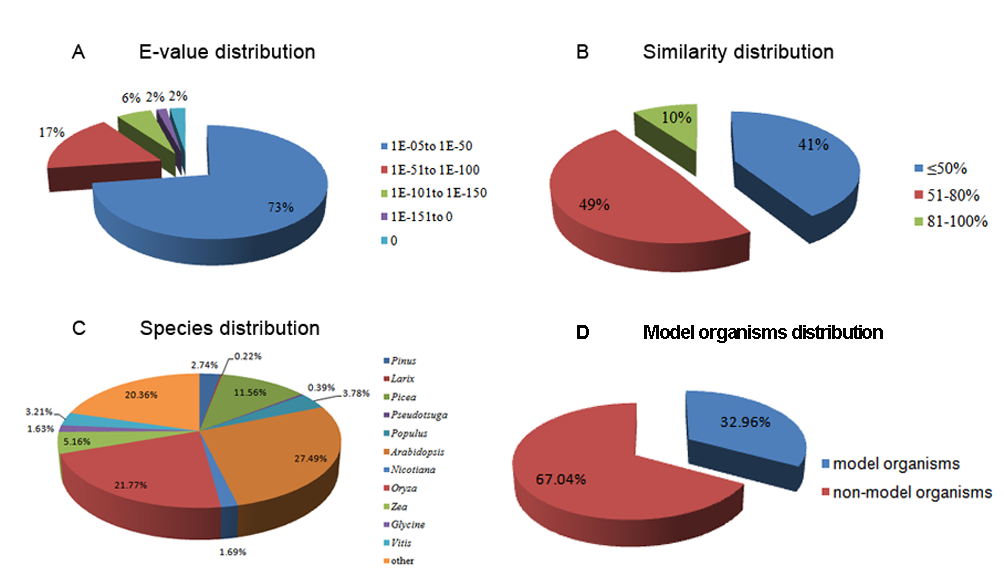

Supplement: Additional file 4 — Characteristics of homology search of Illumina sequences against the Nr databases. (A) E-value distribution of BLAST hits for each unique sequence with a cut-off E-value of 1.0E-5. (B) Similarity distribution of the top BLAST hits for each sequence. (C) Species distribution is shown as a percentage of the total homologous sequences with an E-value of at least 1.0E-5 (we used the first hit of each sequence for analysis). (D) Model organisms distribution is shown as a percentage. [file 1471-2164-14-548-S4.tiff]

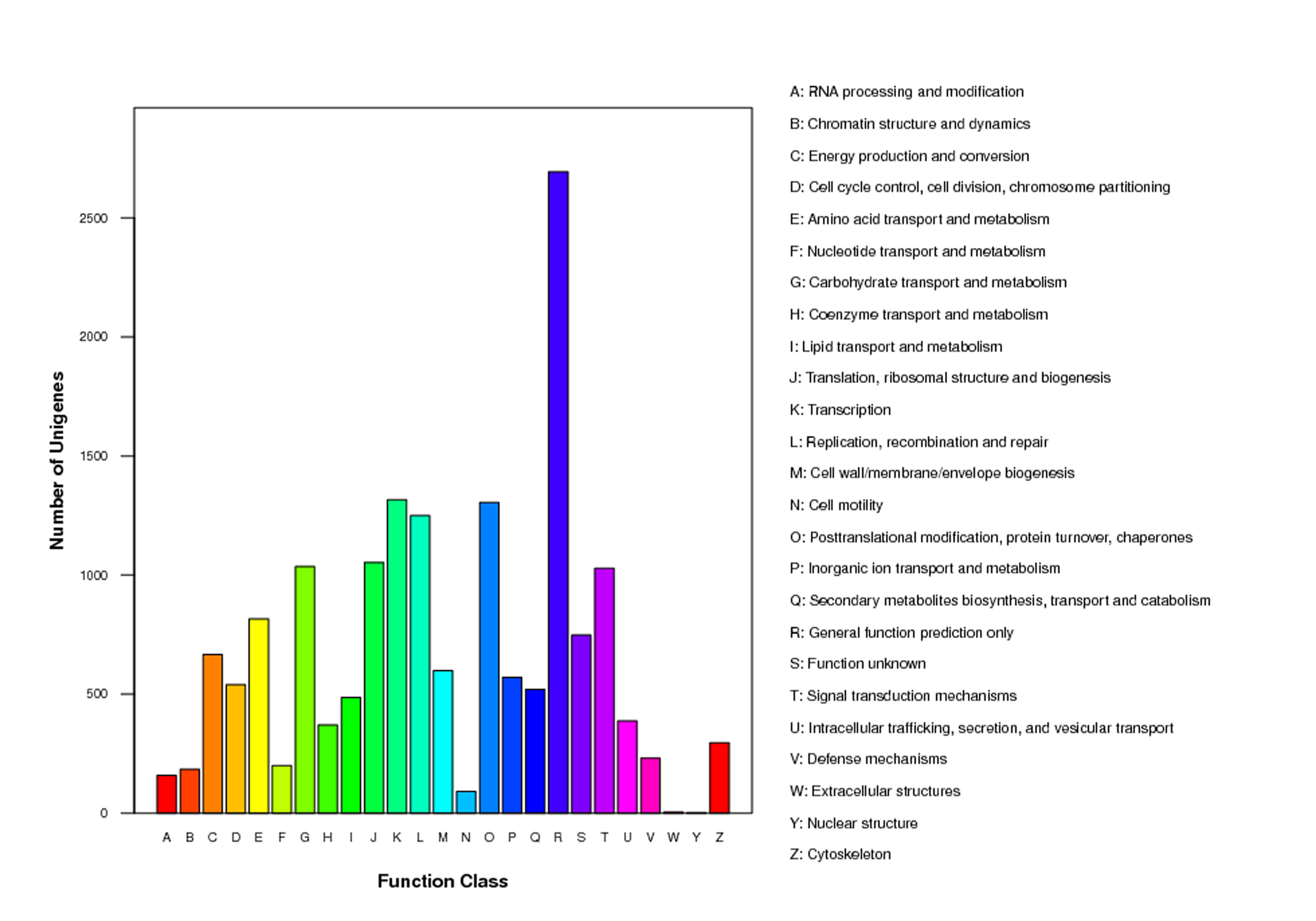

Supplement: Additional file 5 — Histogram presentation of clusters of orthologous groups (COG) classification. These 9,920 sequences have a COG classification among the 25 categories. [file 1471-2164-14-548-S5.tiff]

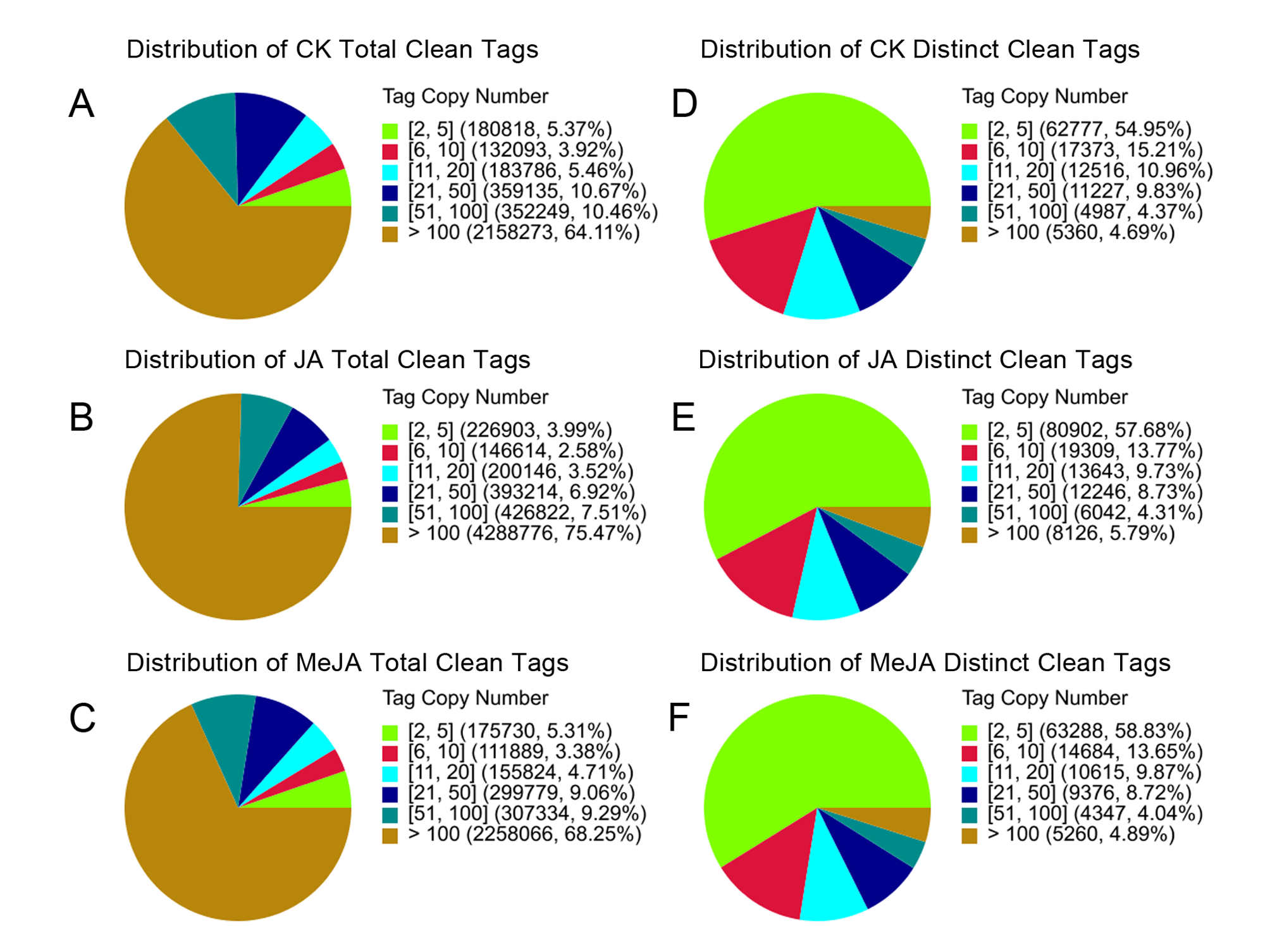

Supplement: Additional file 7 — Distribution of total clean tags (A-C) and distinct clean tags (D-F) over different tag abundance categories. Numbers in the square brackets indicate the range of copy numbers for a specific category of tags. For example, [2,5] means all the tags in this category have 2 to 5 copies. Numbers in the parentheses show the total tag copy number for all tag types in that category. [file 1471-2164-14-548-S7.tiff]

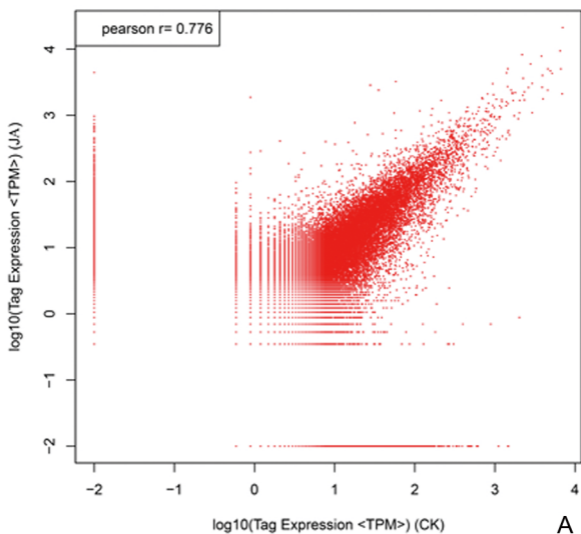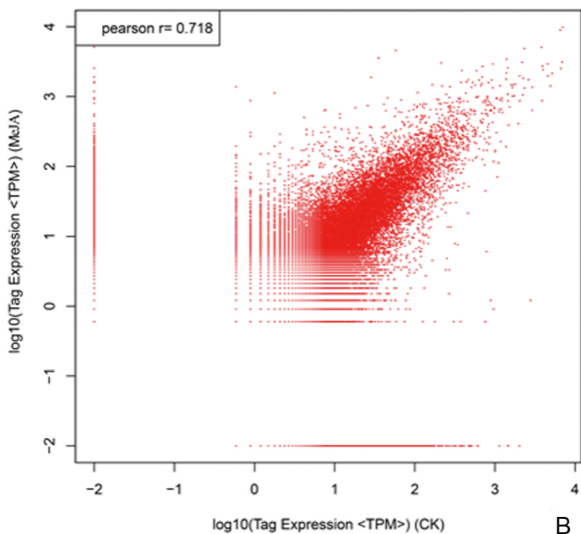

Supplement: Additional file 8 — Correlation analysis of DGE libraries. The correlation between CK vs. JA and CK vs. MeJA libraries are shown. Dots in the figures indicate individual tag entities. Pearson correlation coefficients are shown in the upper left corner of each plot. [file 1471-2164-14-548-S8.pdf]

# CK

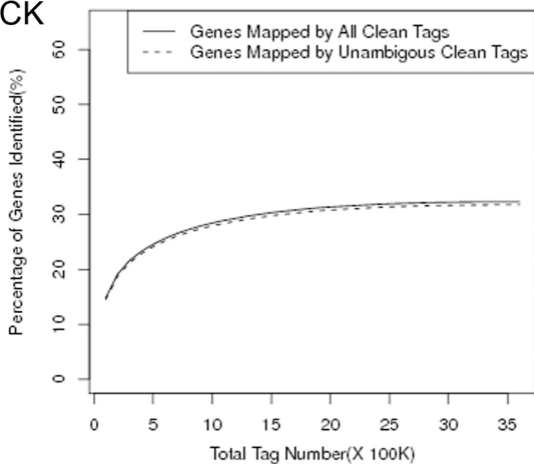

# JA

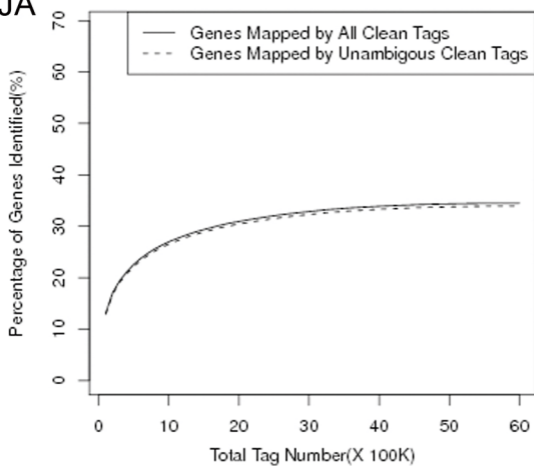

# MeJA

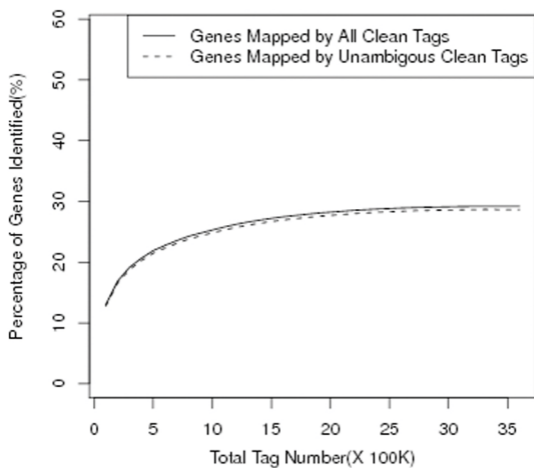

Supplement: Additional file 9 — Relationship between the number of detected genes and sequencing amount (total tag number). All figures show a trend of saturation. When the sequencing amount reaches 3 million, the number of detected genes almost ceases to increase. [file 1471-2164-14-548-S9.pdf]

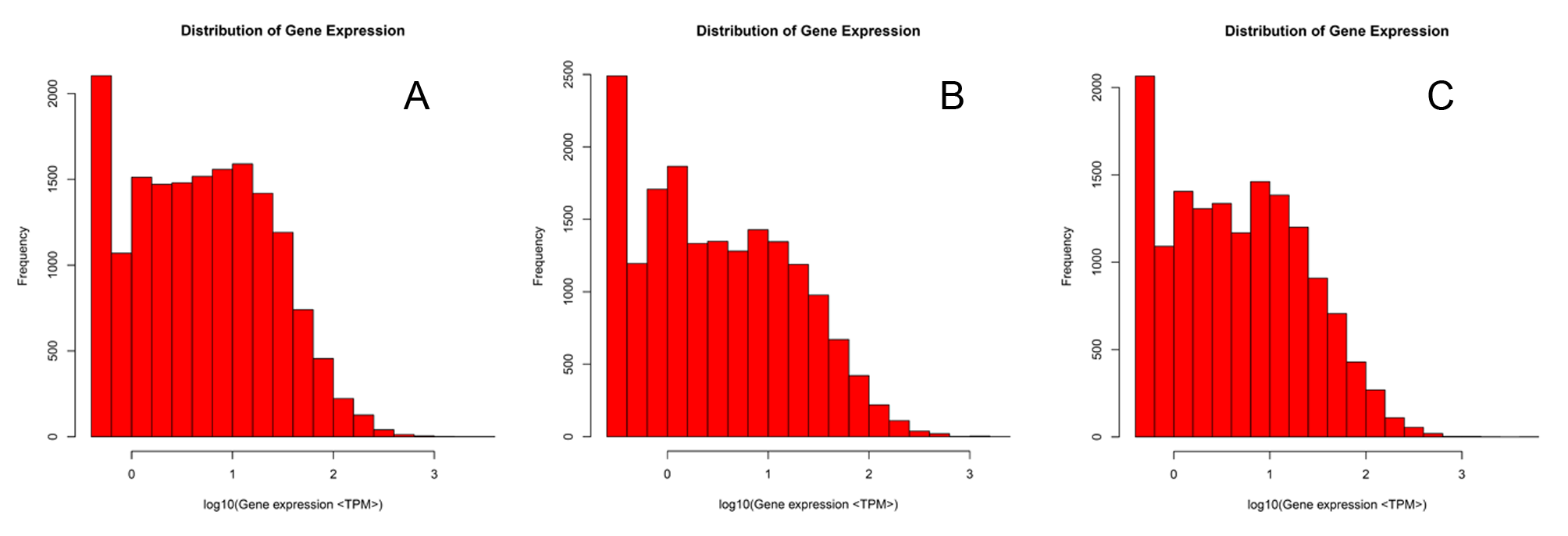

Supplement: Additional file 10 — Level of gene expression for each gene. Gene expression level was determined by calculating the number of unambiguous tags for each gene and then normalizing to TPM (transcript copies per million tags). (A) control solutions CK; (B) JA-treatment; (C) MeJA-treatment. [file 1471-2164-14-548-S10.tiff]

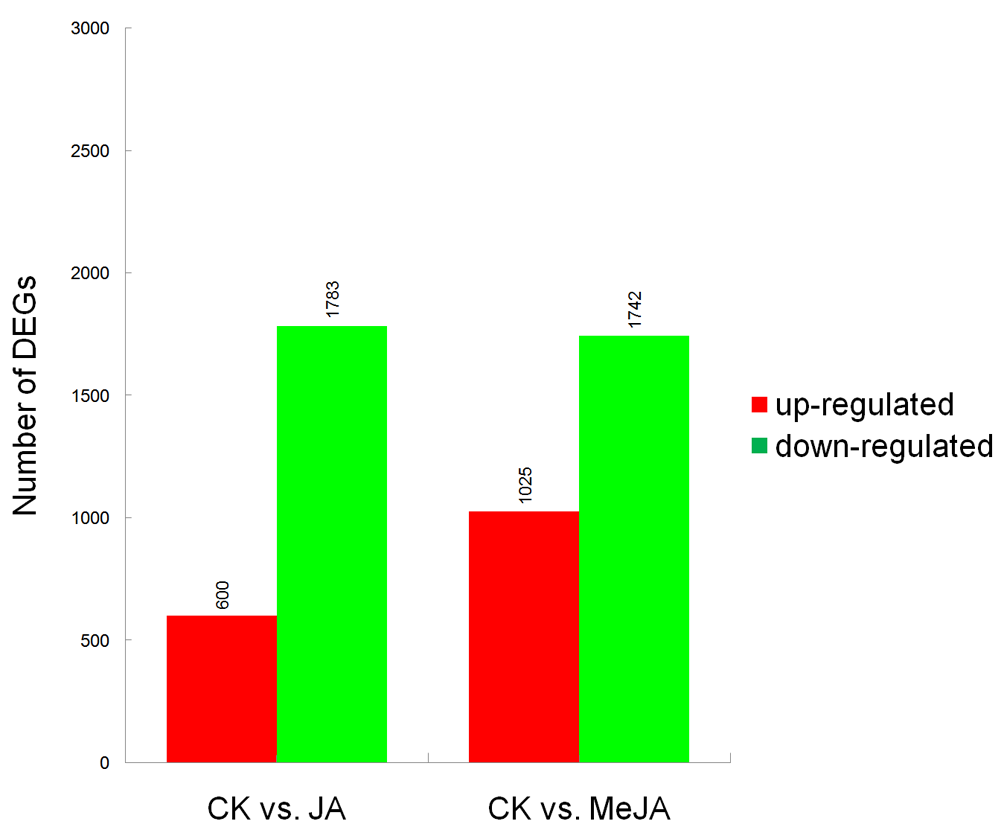

Supplement: Additional file 11 — Changes in gene expression profile induced by different elicitor treatments. The number of up-regulated and down-regulated genes between CK and JA; CK and MeJA is summarized. [file 1471-2164-14-548-S11.tiff]
