# Supplementary material for: De novo characterization of Larix gmelinii (Rupr.) Rupr. transcriptome and analysis of its gene expression induced by jasmonates
Source: BMC Genomics. 2013 Aug 13;14:548. doi: 10.1186/1471-2164-14-548 (PMC3765852; doi:10.1186/1471-2164-14-548)
Supplement: Additional file 6 — Length distribution of Larix gmelinii unigene for CDS predicted via BLAST and ESTScan. (A) Length distribution of Larix gmelinii Unigene. BLAST. cds. fa; (B) Length distribution of Larix gmelinii Unigene. ESTScan. cds. fa. [file 1471-2164-14-548-S6.pdf]

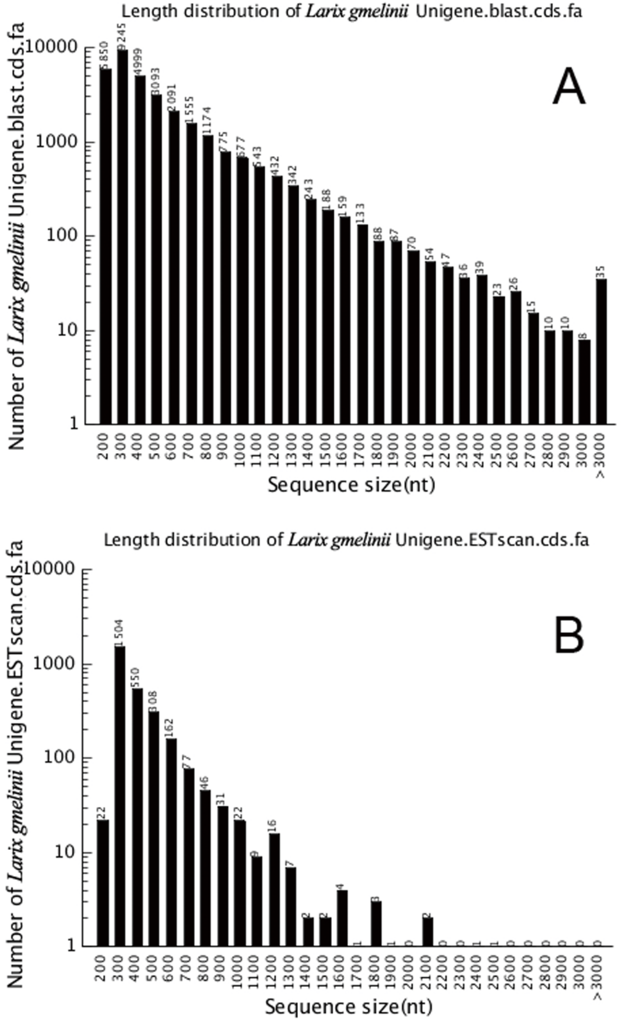

Length distribution of *Larix gmelinii* Unigene.blast.cds.fa
A
Number of *Larix gmelinii* Unigene.blast.cds.fa
10000
1000
100
10
1
5850
9245
4999
3093
2091
1555
1174
775
677
543
432
342
243
188
159
133
88
87
70
54
47
36
39
23
26
15
10
10
8
35
200 300 400 500 600 700 800 900 1000 1100 1200 1300 1400 1500 1600 1700 1800 1900 2000 2100 2200 2300 2400 2500 2600 2700 2800 2900 3000 >3000
Sequence size(nt)
Length distribution of *Larix gmelinii* Unigene.ESTscan.cds.fa
B
Number of *Larix gmelinii* Unigene.ESTscan.cds.fa
10000
1000
100
10
1
22
1504
550
308
162
77
46
31
22
9
16
7
2
2
4
1
3
1
0
2
0
0
1
1
0
0
0
0
0
0
200 300 400 500 600 700 800 900 1000 1100 1200 1300 1400 1500 1600 1700 1800 1900 2000 2100 2200 2300 2400 2500 2600 2700 2800 2900 3000 >3000
Sequence size(nt)
