# Supplementary material for: De novo characterization of Larix gmelinii (Rupr.) Rupr. transcriptome and analysis of its gene expression induced by jasmonates
Source: BMC Genomics. 2013 Aug 13;14:548. doi: 10.1186/1471-2164-14-548 (PMC3765852; doi:10.1186/1471-2164-14-548)
Supplement: Additional file 16 — Significantly enriched GO terms in DEGs. GO terms with corrected-pvalue≤0.05 are significantly enriched in DEGs. [file 1471-2164-14-548-S16.pdf]

Significantly enriched GO terms in DEGs

Ontology	CK vs. JA<br>Gene Ontology term	Corrected P-value	CK vs. MeJA<br>Gene Ontology term	Corrected P-value
cellular component	cytoplasmic part	4.42e-06	plastid part	0.00022
	cytoplasm	5.03e-06	cytoplasm	0.01486
	plastid thylakoid	9.74e-06	plastid	0.01603
	organelle subcompartment	1.07e-05	cytoplasmic part	0.01701
	plastid part	1.48e-05	chloroplast	0.02335
	chloroplast	1.75e-05	plastid envelope	0.02371
	thylakoid	2.58e-05	chloroplast part	0.03330
	chloroplast part	0.00017		
	chloroplast thylakoid	0.00019		
	plastid	0.00023		
	organelle part	0.00045		
	intracellular organelle part	0.00068		
	plastid stroma	0.00377		
	intracellular part	0.00846		
	intracellular	0.00913		
	thylakoid part	0.00918		
molecular function	oxidoreductase activity	0.08915	oxidoreductase activity	0.00045
biological process	-	-	cellular cell wall organization	0.02484

GO terms with corrected-pvalue≤0.05 are significantly enriched in DEGs.
