# Supplementary material for: De novo characterization of Larix gmelinii (Rupr.) Rupr. transcriptome and analysis of its gene expression induced by jasmonates
Source: BMC Genomics. 2013 Aug 13;14:548. doi: 10.1186/1471-2164-14-548 (PMC3765852; doi:10.1186/1471-2164-14-548)
Supplement: Additional file 18 — Differential gene expression of Phenylpropanoid biosynthesis. + means up-regulated; - means down-regulated; log2Ratio: multiples of differentially expressed. [file 1471-2164-14-548-S18.pdf]

Differential gene expression of Phenylpropanoid biosynthesis

	CK vs. JA				CK vs. MeJA			
	Gene ID	Nr annotation	Swissprot annotation	log2Ratio	Gene ID	Nr annotation	Swissprot annotation	log2Ratio
Phenylalanine ammonialyase	Unigene 46956	phenylalanine aminomutase [*Taxus wallichiana* var. *chinensis*]	Phenylalanine ammonia-lyase [*Pinus taeda*]	+4.8	Unigene 46956	phenylalanine aminomutase [*Taxus wallichiana* var. *chinensis*]	Phenylalanine ammonia-lyase [*Pinus taeda*]	+5.7
Trans-cinnamate 4-monooxygenase	Unigene 47342	cinnamate 4-hydroxylase [*Ipomoea batatas*]	Trans-cinnamate 4-monooxygenase [*Glycyrrhiza echinata*]	-1.1	Unigene 47342	cinnamate 4-hydroxylase [*Ipomoea batatas*]	Trans-cinnamate 4-monooxygenase [*Glycyrrhiza echinata*]	-1.3
coumarate 3-hydroxylase	Unigene 42617	coumarate 3-hydroxylase [*Pinus taeda*]	Cytochrome P450 98A2 [*Glycine max*]	-2.5	Unigene 42617	coumarate 3-hydroxylase [*Pinus taeda*]	Cytochrome P450 98A2 [*Glycine max*]	+1.9
Coniferyl-aldehyde dehydrogenase	Unigene 43337	cytosolic aldehyde dehydrogenase [*Oryza sativa* Japonica Group]	Aldehyde dehydrogenase family 2 member B7, mitochondrial [*Arabidopsis thaliana*]	-1.3	Unigene 43337	cytosolic aldehyde dehydrogenase [*Oryza sativa* Japonica Group]	Aldehyde dehydrogenase family 2 member B7, mitochondrial [*Arabidopsis thaliana*]	-1.1

+ means up-regulated; - means down-regulated，log2Ratio: multiples of differentially expressed.
