# Supplementary material for: De novo characterization of Larix gmelinii (Rupr.) Rupr. transcriptome and analysis of its gene expression induced by jasmonates
Source: BMC Genomics. 2013 Aug 13;14:548. doi: 10.1186/1471-2164-14-548 (PMC3765852; doi:10.1186/1471-2164-14-548)
Supplement: Additional file 19 — qPCR primer sequences. [file 1471-2164-14-548-S19.pdf]

Supplementary table 20. qPCR primer sequences

Gene	Forward primer (5'- 3')	Reverse primer (5'- 3')
Phenylalanine ammonialyase	GTACAAGTTTGTGAGGGAAGAATTG	ATTACAGGACATCCATAGCTTAGC
Cinnamate 4-hydroxylase	AGAGGTTCTTGGAAGAGGAGGCC	CACCTTTCTCTGCGGTGTCTATCTT
4-coumarate-CoA ligase	CTTCCAAAGGGCGTAATGCTAAC	GCAGAGGAGAACAGAATTGAGAG
Trans-cinnamate 4-monooxygenase	CAAAGATTGACACCTCAGAGAAAG	GCTCCCTTTGTATCATCATTATCC
Caffeic acid 3-O-methyltransferase	GGGAGTCGTGCACATTGATGTTATC	GACTTGAGCCTTAGTTCTTGCGGAA
Caffeoyl-CoA O-methyltransferase	GGAGGTAACAGCAATAGACAAAGATC	GTGATAGTGCTTGTAGCTTGACTTG
